# Defective Medical Education

**Published:** 1898-01

**Authors:** 


### EDITORIAL.

The office of THE JOURNAL is 311 and 312 Fitten Building.

Address all communications, and make all remittances payable to THE ATLANTA MEDICAL AND SURGICAL JOURNAL, Atlanta, Ga.

Reprints of articles will be furnished, when desired, at a small cost. Requests for the same should always be made *on the manuscript*.

### DEFECTIVE MEDICAL EDUCATION.

The above is the title of a recent editorial in the *New York Medical Record*. The editor says: "The epidemic of yellow fever in the South may turn out, after all, to be not an unmixed evil. If by its means more attention should be paid to the study of bacteriology, and the etiology of yellow fever should be made clear, then it may be said that the good it will have done will outweigh the evil." Looking at this subject from a scientific standpoint, there are few who would not agree with the statements above, and yet, from a humanitarian and business point of view, there would certainly be a lack of unanimity, especially from the people of the South. In the first place we do not think that the presence of yellow fever in the South will add much information to our present knowledge of its etiology, for the disease caused such widespread consternation in those places and cities where it occurred that time was not given to its scientific aspect so much as effort on the part of neighboring towns to institute a "shotgun quarantine." Yellow fever, except in a few exotic cases, is a rare occurrence in the South, and medical health officers at our seaport towns bear out the observation that when cases do occur they are usually of foreign growth. It is true that when the fever obtains a foothold in some of our Southern cities its tendency is to spread, especially if the environments are such as favor zymotic diseases, but epidemics of yellow fever come in waves, and fortunately this goodly country of ours is not often visited by such a pestilential mishap. This last epidemic of fever shows conclusively one thing, and that is that either the type of the disease was much milder than those of previous occurrence, or that the sanitary regulations of our Southern cities have greatly improved. The latter is certainly the more plausible explanation, for as the South has advanced in her material aspects, so also she has made rapid progress in her medical education. While it is true that there are too many medical colleges for the good of the medical profession, it is equally true that the South is not near so much crowded with these institutions as some other portions of our country. How long this will continue to be the case we are unable to say, since so-called medical colleges are springing up like mushrooms all over the country. The Southern colleges offer to Southern students of medicine many advantages which they can not obtain in the North. The Southern cities like Nashville, Atlanta, Charleston, Augusta, New Orleans, where excellent medical schools are located, are all large enough to furnish most excellent clinical material. The rare cases in both medicine and surgery which a student sometimes sees in the hospitals of some of our big Northern cities are not the ones which they will have to treat when they begin the practice of medicine. The *Hospital*, an English publication, in an editorial recently calls attention to a speech made by the dean of St. George's Hospital, in which he referred to some of "the defects in the system of modern medical education," and particularly the want of knowledge in Great Britain of the tropical diseases. These statements are certainly applicable to the study of medicine in the United States. It is a well known fact that certain diseases are peculiar to certain localities, and it is equally true that geographical positions change the character of the same disease. The young man who expects to practice medicine in the South cannot expect to become familiar with Southern fevers in Northern hospitals. Do not understand us as meaning that a young man should narrow his medical education to one locality, for a man's knowledge was never too extensive. What we do mean is that our Southern colleges are every year better equipped for turning out good physicians, and their hospitals offer a far better inducement for the study of those diseases which they will meet with in their everyday practice than can be found in many of the Northern institutions. We believe the day is rapidly coming when Southern young men will seek Southern colleges in their efforts to gain a medical education. R.

